# Indigenous Medicinal Plants

**Published:** 1864-06

**Authors:** W. T. Grant

**Affiliations:** P. A. C. S.


Art. III.—*Indigenous Medicinal Plants.* By Assistant Surgeon W. T. Grant, P. A. C. S.

If it is not presumptuous in me, I will ask a small space in your journal that I may call the attention of the profession to a few of our indigenous medicinal plants. I am bold enough to express the opinion that some of these plants now mentioned have never before been brought to the notice of the medical public in any way whatever. And should the good opinion which I have formed of some of them be confirmed by experience, I shall be well repaid for the labor which I have bestowed upon this branch of science. I tender the following observations, also, with the supposition that you will perhaps but rarely have contributions upon this or kindred subjects. And, perhaps, in consequence of this, my mite may be kindly received.

I will not weary your patience, nor impose upon the kindness of your readers with a desertation upon the well-known virtues of cornus Florida, liriodendron tulipifera, eupatorium perfoliatum, smilax sarsaparilla, the salicidæ, and gelseminum semper virens, nor the different species of rubus and quercus, but will attempt to show new virtues in other well-known plants, or valuable virtues in plants hitherto unknown to the profession.

I cannot, however, neglect to mention the existence among us of the following old medicinal plants, the extracts from which have long been used: taraxacum dens-leonis, and datura stramonium. They are quite common, and may be found in nearly all the Confederate States. The leaves of the datura stramonium, when dried and smoked in an ordinary pipe, afford great relief in asthma. I must also mention the following plants: sanguinaria canadensis and lobelia inflata, asclepias tuberosa, aristolochia serpentaria, spigelia marilandica, melia azedarach, podophyllum peltatum and polygala senega.

The palma christi, from the seeds of which is expressed the castor oil, can be grown to any extent with us.

A decoction of the leaves of sambucus canadensis, (common elder) answers excellently as a disinfectant, and is a safe application for offensive sores, wounds, &c.

We have quite a number of species of gentian, all of which perhaps possess tonic virtues.

I know of no more soothing, agreeable collyrium than an infusion of the young branches of ulmus fulva, (elm), or especially of laurus sassafras, (common sassafras). And the excellence of the domestic remedy hoarhound, (marrubium vulgare), in catarrhal affections, is well known.

The genus smilax, in addition to furnishing us with the fine alterative sarsaparilla, also affords a good remedy for diarrhœas and dysenteries. This is smilax pirmila, (Darby), and is used in strong decoctions.

I am well satisfied that a strong decoction of any part of ilex opaca, taken internally, will prove eminently useful, if not positively curative, in every variety of dropsy. It grows in all parts of Georgia.

And I think that by cultivation, any species of our common poppy could be made to produce opium. Somewhat over a year since, I saw a specimen of opium thus made, (a few grains only), and I supposed from its odor and appearance that it was equal to any from the East.

Our efforts to procure a reliable substitute for quinine, has thus far nearly proved a failure, unless the turpentine, as recently recommended in this journal, should answer; or common salt, (chloride of sodium), which has been very highly recommended to me. Indeed, we may very well believe that salt possesses good powers, as we know how excellent a remedy it is in enlarged spleen, "fever cake," which results from long continued intermittents. I have, to a limited extent, used the berries of cornus Florida, and also a compound decoction of the barks of cornus Florida, liriodendron tulipifera, and prunus virginiana, alias cerasus serotina, (wild cherry), and with very fair success. The "Georgia bark," (pinkneya pubens), among plants, seems to me to be entitled to the first place as a substitute for quinine, although I have used it but little. It grows very abundantly in lower Georgia. I will take the liberty of sending some of it to Prof. Jos. Jones, with the request that he will analyze it and publish the result.

Cephalanthus occidentalis, (button bush), which is also very abundant in lower Georgia, is esteemed very highly both in and out of the profession, as an excellent remedy in catarrhs, &c. I sometimes think that in combination with the wild cherry, it would be almost competent to arrest incipient tubercular consumption.

I shall now speak more fully of some three or four other plants, which I do not think have ever been mentioned by any writer.

The gordonias lasyanthus (Wood) grows quite abundantly in the tide-water districts of Georgia, and perhaps of other States. I have never analyzed it, and am not, therefore, very well acquainted with it. Its common name is loblolly bay. The bark of the roots, or stem, makes one of the finest possible poultices for certain conditions of wounds, &c. I have seen its effects, where, without it, I cannot see how extensive sloughing could have been avoided. I have heard the remark of an old practitioner to the effect that "nothing else need be used, if this fails, to arrest mortification." It seems to possess stimulating and other virtues which render it very valuable as an application to wounds having a tendency to mortification. Of course it would be an excellent application when it is desirable to excite a healthy suppuration.

Platanthera stricta, (habenaria cristata, Darby,) is an herbaceous plant very common in lower Georgia, and possesses curative virtues in the bite of the rattlesnake. Its common name here is rattlesnake's master. (There are several other plants all having this common name.) I have no doubt that it has some virtues of the kind from the abundant evidence furnished me by the people. Hunters cure their dogs with it when bitten. I have never seen it tested, and only mention it that it may be tried by others should the opportunity be presented. I may mention, as an evidence of the faith which some here have in it, that they firmly believe that a rattlesnake will not bite a man at all if he has only some of the root in his pocket.

Another remedy for the bite of the rattlesnake is the baptisia perfoliata, which is also very abundant here. I will only add the following evidence of its virtues, which is a statement taken from a letter to me from my esteemed friend, Prof. W T. Fray, than whom there is no finer botanist in the South: "A gentleman in Florida had a negro woman bitten by a rattlesnake, and a physician was sent for at once. In the meantime a neighbor recommended that a decoction of the root of this plant should be used. It was done, and when the physician arrived the negro woman had entirely recovered."

In private practice, some years back, my attention was called to a plant, whose good effects in epilepsy, as reported, were extraordinary. And such wonderful cures were vouched for by intelligent citizens, that I determined to see for myself. And having an epileptic on hand, I administered the remedy. For two months during the time I used it, no paroxysm occurred, although they were frequent before. At the end of this time the patient went away and I saw no more of him. I also gathered a quantity of the roots and sent them to other physicians with the request that they should test it; but for some reason or other I never heard from any except one package. And the answer to this was a request to send more, that it did well. This is the extent of my practical knowledge of its utility as a remedy. But I have confidence to believe that it will be found an excellent remedy for epilepsy. And if so, why not in other neurotic diseases, as well—hysteria, chorea, tetanus, &c.?

I am unfortunate in not being able to give the exact name of this plant. I analyzed it at the time, and made it vernonia fasiculata, but that analysis is not reliable. I now know that this vernonia is not a native of the South. And if it is a vernonia stalk at all, it is oligaphylla. But I am now quite well satisfied, although I have not seen it since, that it is a liatris, and most probably, spicata. If I could see it now I could determine it at once. It grows abundantly in Richmond, Columbia, and Warren counties, in Georgia, and I think is very widely diffused through the South.

I have just received No. 3 of this journal, containing Surg. W. A. Carrington's report of eruptive fevers, &c. In the latter part of this report he states that the saracenia purpurea has been recently used in small pox, and gave evidence of great future usefulness in that dire disease. Although it grows in Georgia, I have not seen *this* species of saracenia, as my opportunities for botanizing are limited. But I find here its cogeners, saracenia flava and variolaris, in the greatest abundance. And from the great similarity in habits, size, general appearance, and every thing that obtains among the species of this genus, I am inclined to think that a like similarity must extend to their therapeutics; and that if purpurea is valuable as a remedy in any particular disease, flava and variolaris and the other species would fulfill the same purpose, and equally as well.

